# Supplementary material for: Structures and membrane interactions of native serotonin transporter in complexes with psychostimulants
Source: Proc Natl Acad Sci U S A. 2023 Jul 12;120(29):e2304602120. doi: 10.1073/pnas.2304602120 (PMC10629533; doi:10.1073/pnas.2304602120)
Supplement: Supplementary file 1 — Appendix 01 (PDF) [file pnas.2304602120.sapp.pdf]

PNAS

Supporting Information for

Structures and membrane interactions of native serotonin transporter in complexes with psychostimulants

Dongxue Yang[1,4], Zhiyu Zhao[3], Emad Tajkhorshid[3], and Eric Gouaux[1,2,*]

1 Vollum Institute, Oregon Health & Science University, Portland, Oregon 97239, USA.

2 Howard Hughes Medical Institute, Oregon Health & Science University, Portland, Oregon 97239, USA.

3 Department of Biochemistry, NIH Center for Macromolecular Modeling and Bioinformatics, Beckman Institute for Advanced Science and Technology, and Center for Biophysics and Quantitative Biology, University of Illinois at Urbana-Champaign, Urbana, Illinois 61801, USA.

4 Present address: Department of Urology, Institute of Urology (Laboratory of Reconstructive Urology), West China Hospital, Sichuan University, Chengdu, Sichuan, China.

*Eric Gouaux

Email: gouauxe@ohsu.edu

This PDF file includes:

Figures S1 to S8
Table S1

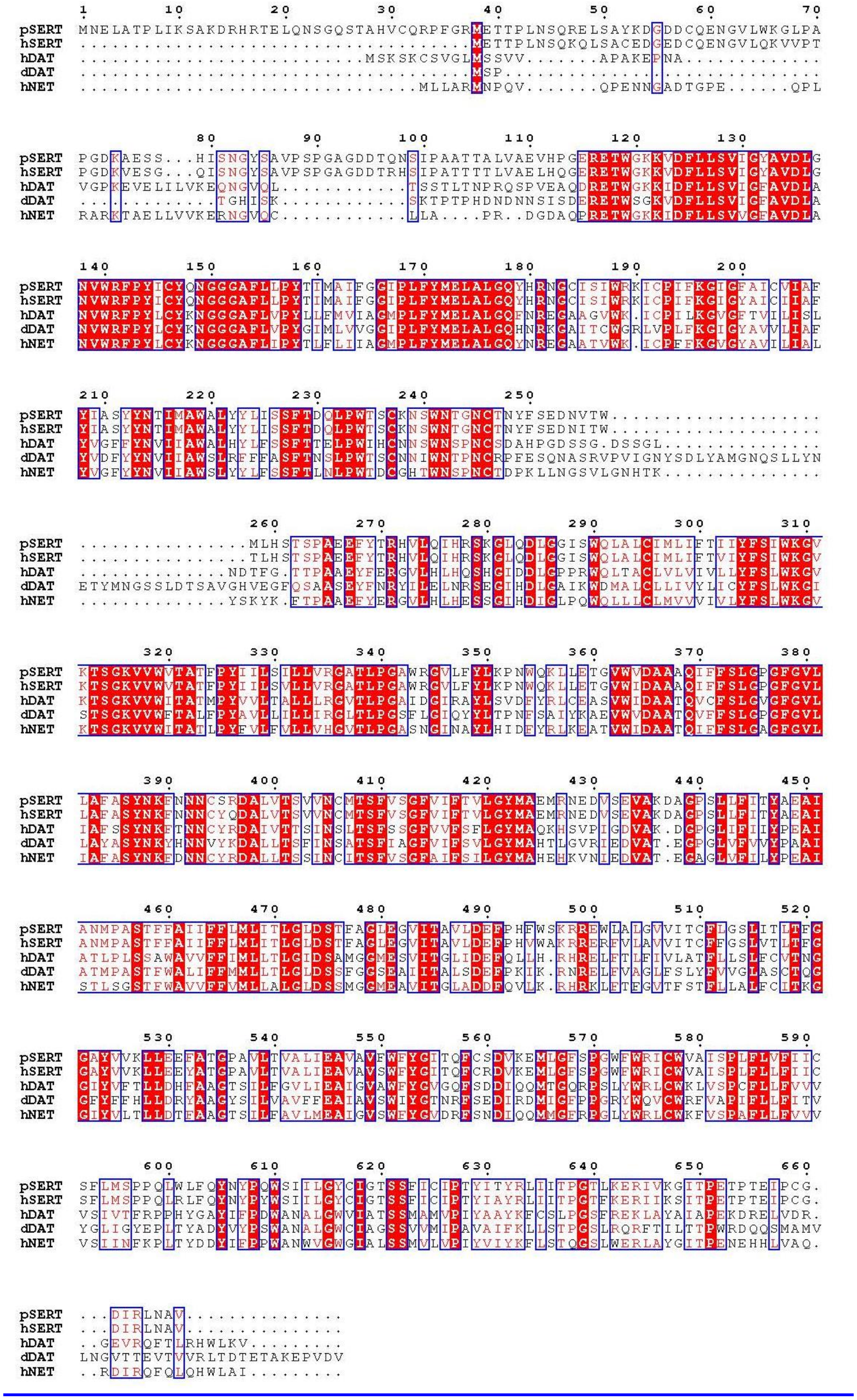

Figure S1. Multiple sequence alignment of SERT, DAT and NET orthologues. Residues in black are not conserved, those in red are conservatively substituted, and those in white with red background are conserved.

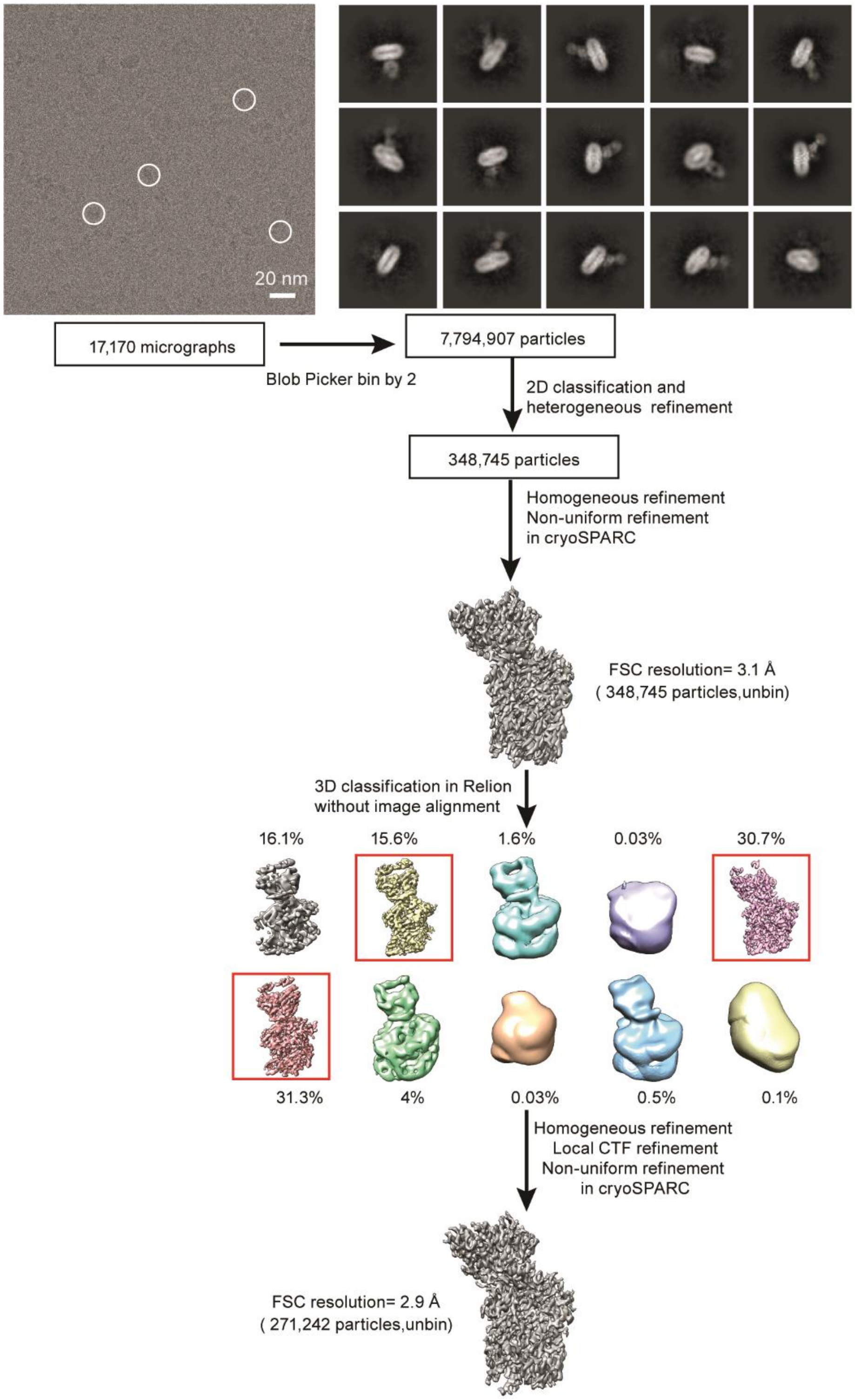

Figure S2. 3D reconstruction of the pSERT-methamphetamine complex. Flow chart for cryo-EM data analysis.

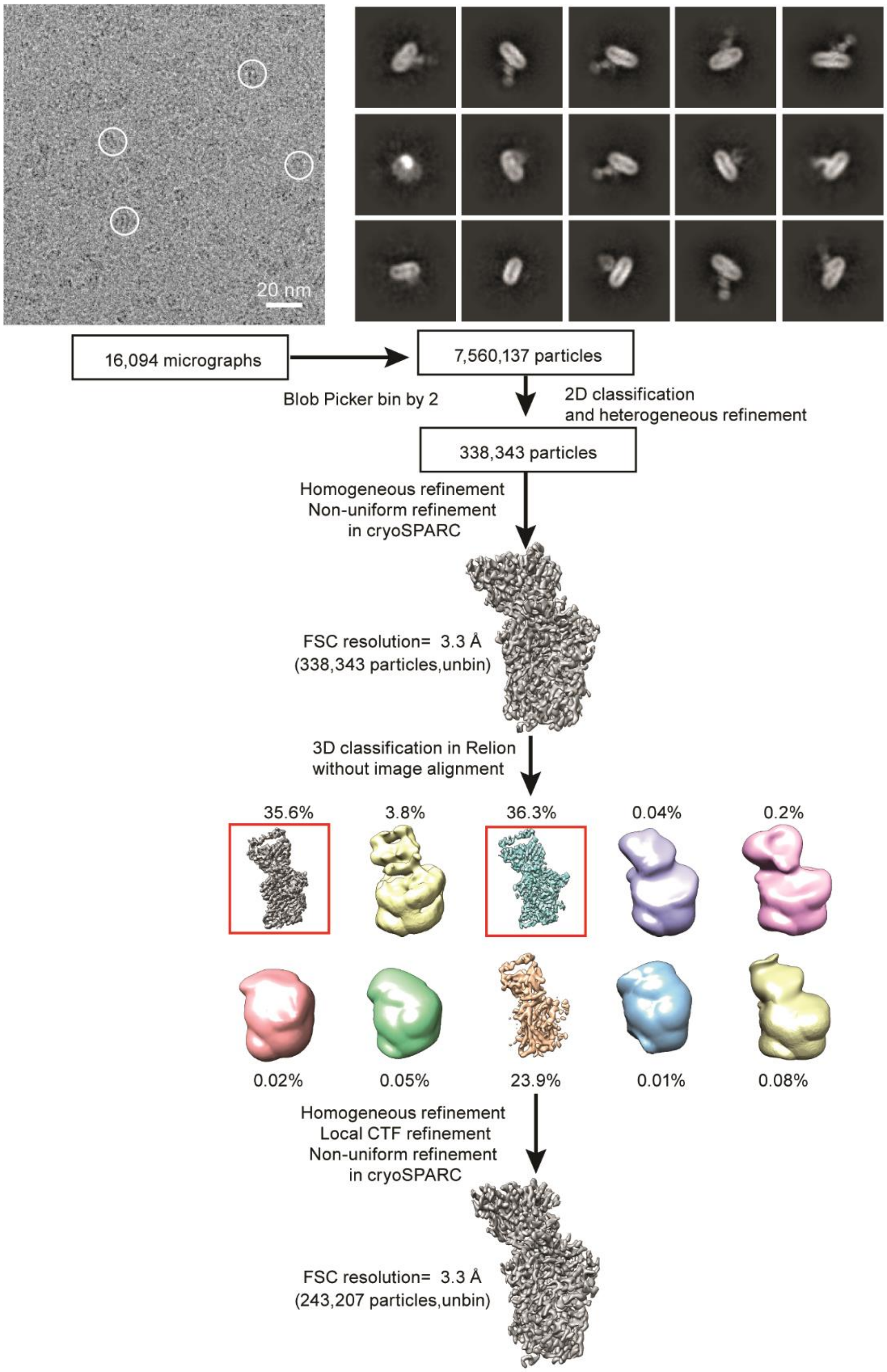

Figure S3. Cryo-EM analysis of pSERT-cocaine complex. Flow chart for cryo-EM data analysis.

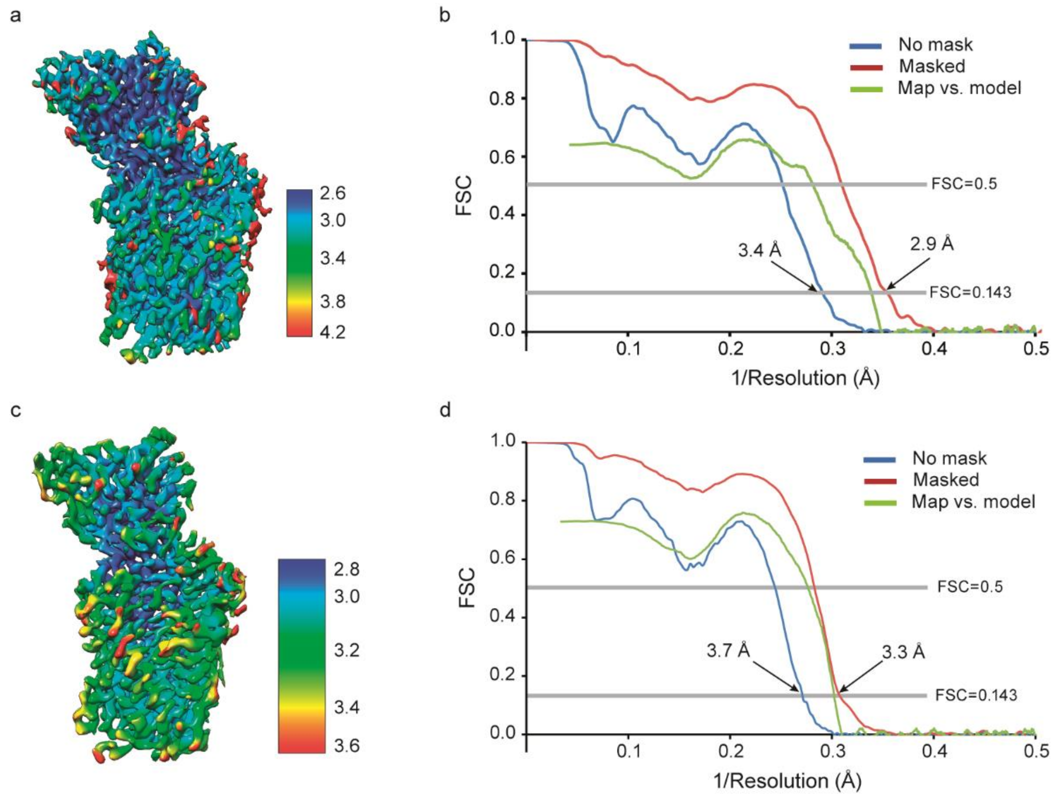

Figure S4. Local resolution maps, overall plotted resolutions, and global map-model agreements. a (+)-methamphetamine-pSERT Fab complex map is colored by local resolution. **b** Fourier shell correlation (FSC) curves for (+)-methamphetamine-pSERT Fab complex. **c** Local-resolution distribution of the cocaine-pSERT Fab map. **d** Map-map and map-model FSC curves for cocaine-pSERT Fab complex.

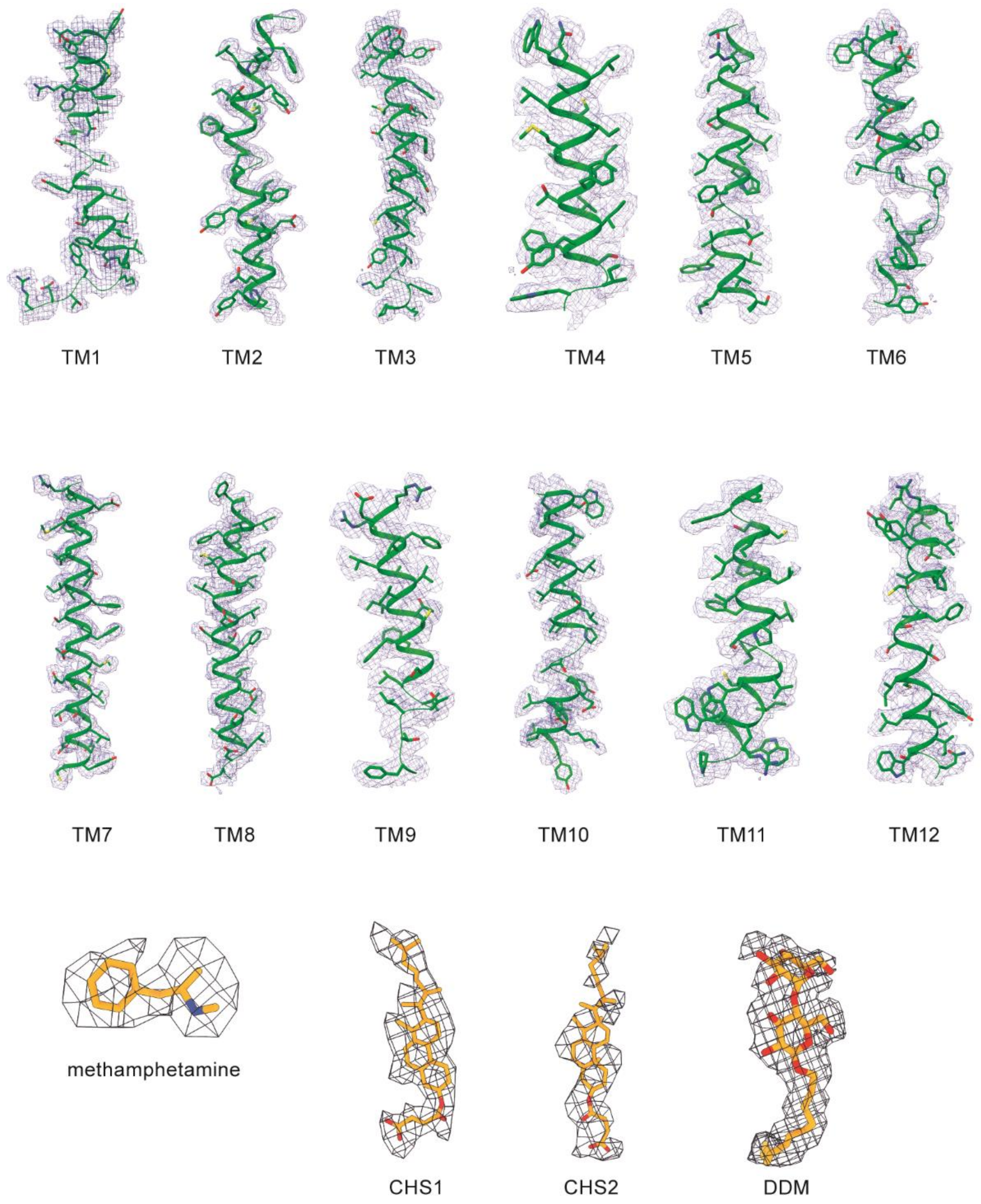

Figure S5. Representative densities of (+)-methamphetamine-pSERT Fab complex. Representative densities for transmembrane helices, (+)-methamphetamine cholesteryl hemisuccinate (CHS), and dodecylmaltoside (DDM).

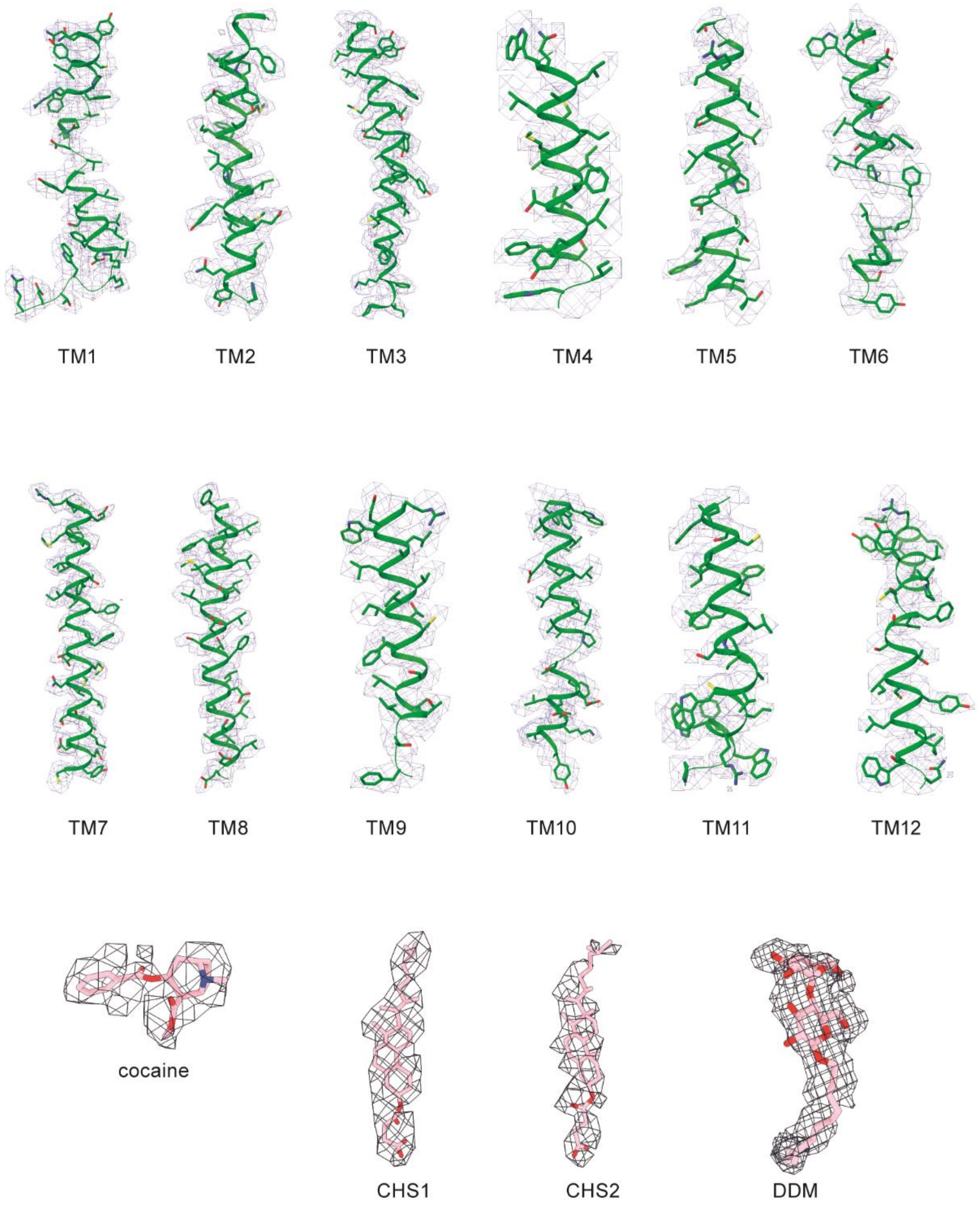

Figure S6. Representative densities of cocaine-pSERT Fab complex. Density fitting of transmembrane helices, cocaine, CHS, and DDM.

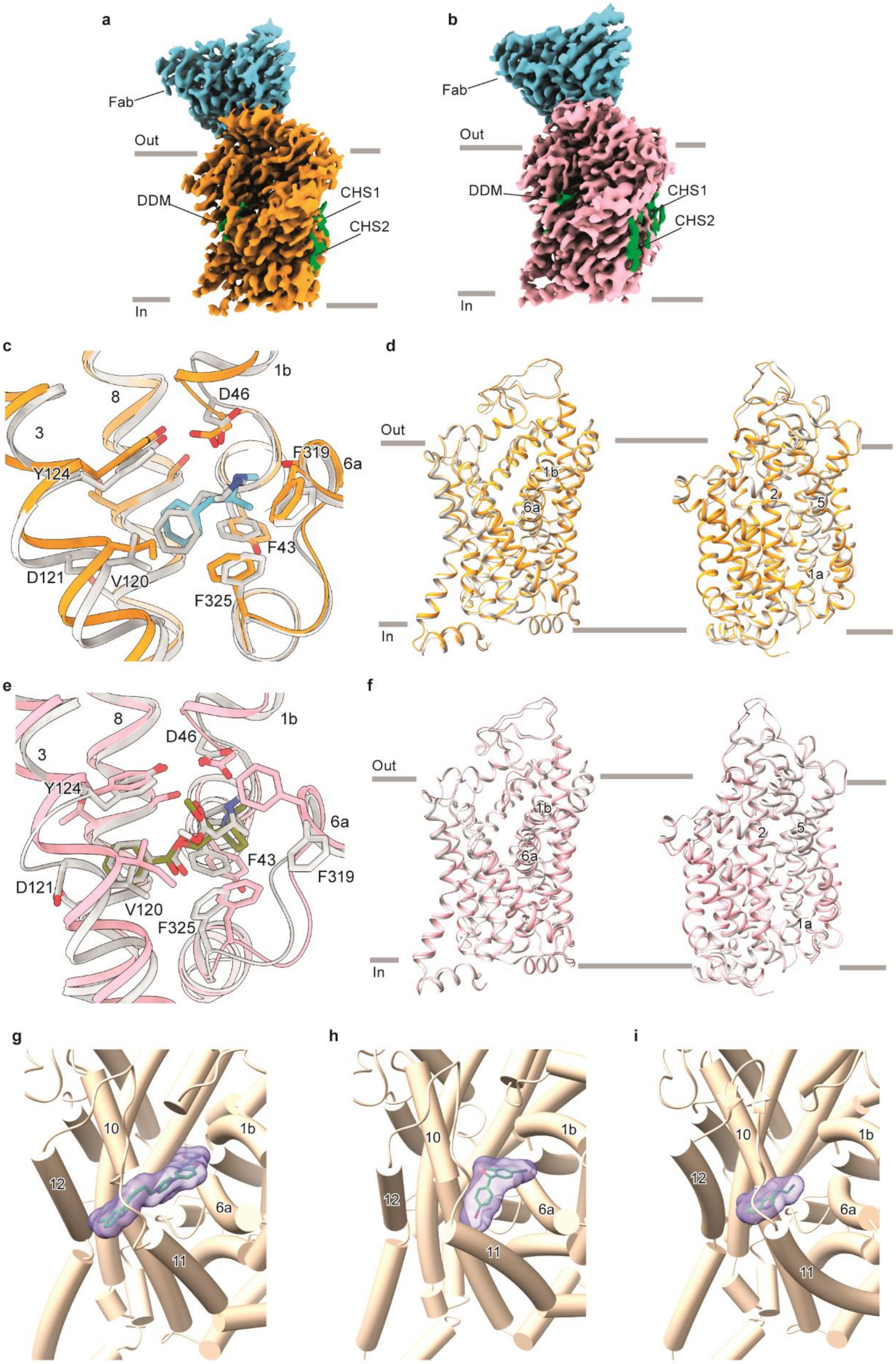
a
Fab
Out
DDM
CHS1
CHS2
In
b
Fab
Out
DDM
CHS1
CHS2
In
c
8
1b
D46
3
F319
6a
Y124
F43
D121
V120
F325
d
Out
1b
6a
2
5
1a
In
e
8
1b
D46
3
Y124
6a
F43
F319
D121
V120
F325
f
Out
1b
6a
2
5
1a
In
g
10
1b
12
6a
11
h
10
1b
12
6a
11
i
10
1b
12
6a
11

Figure S7. The cryo-EM density maps of ligand-bound pSERT Fab complex and comparison of ligand binding in the central sites of SERT with dDAT. a and **b** The cryo-EM density maps of (+)-methamphetamine-pSERT (a) and cocaine-pSERT (b) complex. **c** Superposition of the binding pockets of the (+)-methamphetamine-dDAT structure (PDB code: 4XP6) in grey with binding pockets of (+)-methamphetamine-pSERT (orange). Residues interacting with (+)-methamphetamine in dDAT have been indicated. **d** The superimposed pSERT-(+)-methamphetamine and hSERT-5-HT (outward-facing, PDB code: 7LIA). **e** Superposition of the central binding pocket of cocaine-dDAT structure (PDB code: 4XP4, grey), with central binding site of cocaine-pSERT (pink). Residues interacting with cocaine in dDAT have been indicated. **f** Structural comparison of pSERT-cocaine and hSERT-5-HT (outward-facing, PDB code: 7LIA). **g-h** Occupancy of the allosteric site by vilazodone (g), citalopram (h), and serotonin (i).

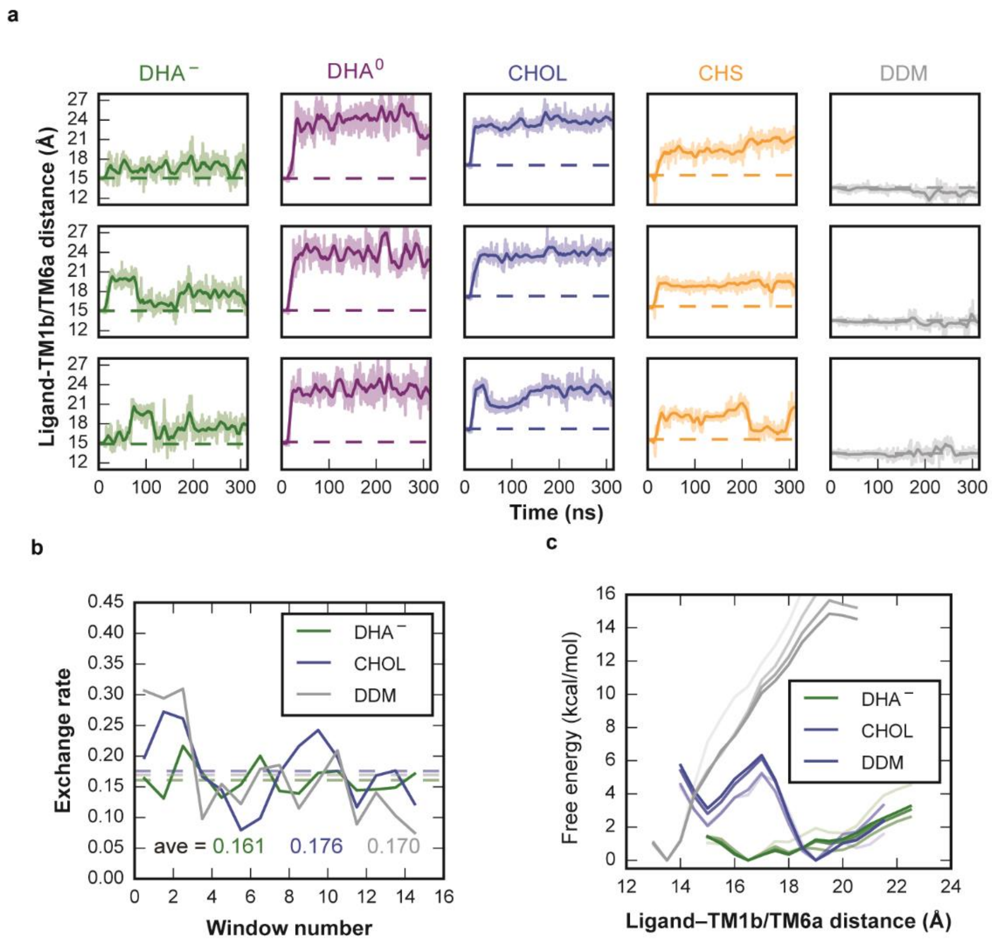

Figure S8. Stability of lipids in the allosteric site monitored by root-mean-square deviation (RMSD) and convergence of BEUS simulations. a Time series of ligand-TM1b/TM6a distance of the ligands at the allosteric site. Data for DHA–, DHA0, CHOL, CHS, and DDM are plotted in green, purple, blue, orange, and gray, respectively, and are shown for the three independent simulations in each case. The ligand-TM1b/TM6a distance is measured as the center-of-mass distance between heavy atoms in the ligand and Cα atoms from TM1b and TM6a (residues 145-148 and 361-364). Dashed lines indicate the initial distances of each molecule. Plots are smoothed using a sliding window of 1 ns. **b** Exchange rates between neighboring windows monitored during the 60-ns BEUS simulations. **c** Convergence of the free energy profiles along the increase in time (10, 30, 50, and 60 ns, indicated by increasing opacities) of the simulations.

Table S1. Supplementary Table 1 Cryo-EM data collection, refinement and validation statistics

	**SERT-methamphetamine** (EMD-) (PDB)	**SERT-cocaine** (EMD-) (PDB)
**Data collection and processing**		
**Magnification**	105,000	
**Voltage (kV)**	300	
**Electron exposure (e–/Å^2)**	60	
**Defocus range (μm)**	-1.0 to -2.5	
**Pixel size (Å)**	0.831	
**Symmetry imposed**	C1	C1
**Initial particle images (no.)**	7,794,907	7,560,137
**Final particle images (no.)**	271,242	243,207
**Map resolution (Å)**<br>**FSC threshold**	2.9<br>0.143	3.3<br>0.143
**Map resolution range (Å)**	4.2-2.6	3.6-2.8
**Refinement**		
**Initial model used (PDB code)**	7LIA	7LIA
**Initial model CC**<br>**Model resolution (Å)**<br>**FSC threshold**	0.628<br>3.4<br>0.5	0.623<br>3.6<br>0.5
**Map sharpening *B* factor (Å^2)**	-95.3	-103.1
**Model composition**		
**Non-hydrogen atoms**<br>**Protein residues**<br>**Ligands (atoms)**	6188<br>769<br>116	6199<br>769<br>127
***B* factors (Å^2)**		
**Protein**<br>**Ligand**	59<br>77	29<br>36
**R.m.s. deviations**		
**Bond lengths (Å)**<br>**Bond angles (°)**	0.002<br>0.631	0.002<br>0.505
**Validation**		
**Refined model CC**<br>**MolProbity score**<br>**Clashscore**<br>**Poor rotamers (%)**	0.680<br>2.15<br>8.76<br>4.35	0.684<br>1.55<br>6.79<br>0.00
**Ramachandran plot**		
**Favored (%)**<br>**Allowed (%)**<br>**Disallowed (%)**	96.85<br>3.15<br>0	96.97<br>3.03<br>0
